# When a dream comes true: birth of the African Crystallographic Association (AfCA)

**DOI:** 10.1107/S2056989023010757

**Published:** 2024-01-05

**Authors:** Andreas Roodt

**Affiliations:** a Department of Chemistry, University of the Free State, Bloemfontein 9300, South Africa; IUCr, United Kingdom, and University of Bari, Italy

**Keywords:** African Crystallographic Association (AfCA), historical notes, outreach, people and events.

## Abstract

This paper summarizes brief perspectives on the historic process of establishing an African Crystallographic Association (AfCA) and includes representative references. It covers activities within four arbitrarily selected, approximate time slots, *i.e.*, 1890s–1999, 2000–2013, 2014–2019 and 2020–2023. A genuine attempt is made to include appropriate role players, organizations and accompanying events within these periods. It concludes with the official admission of AfCA as the fifth Regional Associate of the IUCr at the 26th Congress and General Assembly of the IUCr in Melbourne, Australia in 2023.

## Abstract 2

This paper summarizes brief perspectives on the historic process of establishing an African Crystallographic Association (AfCA), including appropriate role players, organizations and accompanying events. It concludes with the official admission of AfCA as the fifth Regional Associate of the IUCr at the 26th Congress and General Assembly of the IUCr in Melbourne, Australia in 2023.

## 1. Introduction

This paper summarizes brief perspectives on the historic process of establishing an African Crystallographic Association (AfCA), and includes selected, but hopefully representative references. However, it never claims to give detailed early referencing, primarily since these are not readily available, and moreover, it would be too extensive. The paper covers four arbitrarily selected time slots and ends with the official admission of AfCA as the fifth Regional Associate of the IUCr at the 26th Congress and General Assembly of the IUCr in Melbourne, Australia.

In the author’s view, crystallography ‘arrived’ in Africa at different times but in two fairly distinct thrusts, namely, in (i) South Africa, being a British colony and therefore having direct contact with the Bragg group, and (ii) North Africa *via* the principally French/Arabic regions. The latter proceeded somewhat slower than the former.

These are briefly summarized in Section 2 and are considered important to illustrate crystallographic activity in countries across Africa. Up to now, Africa was incorporated under the European Crystallographic Association (ECA), since until 1999 crystallography was only practiced significantly in five African countries, *i.e*., South Africa, Egypt, Tunisia, Algeria, and Morocco. Moreover, by 1999 only Egypt (1978) and South Africa (1950) were members of the IUCr, while Algeria, Cameroon and Morocco joined in 2014 (Haynes, 2023 ▸; IUCr, 2014*a* ▸), and Tunisia in 2017 (IUCr, 2017 ▸).

Table 1 ▸ lists the selected time slots wherein the development of crystallographic activity in Africa are arbitrarily grouped, *i.e*. (i) up to 1999, (ii) 2000–2013, (iii) 2014–2019, and (iv) 2020 onwards. Also indicated therein are some individuals and associations closely related with activities and outcomes. More details on these time slots are discussed stepwise in Sections 3–6.

## 2. Crystallography enters Africa (up to 1999)

### 2.1. South Africa

Mineralogical/Geological activity was, as expected, at the forefront earlier due to the never-ending quest for valuable minerals and gemstones. To this effect Geology in South Africa (SA) was already organized in the 1890s (GSSA, 1896 ▸), with the first talk presented by Draper (1896 ▸). Mineralogy was formally organized only some five decades later.

The appointment of Alexander Ogg as professor in Physics in 1905 at the Rhodes University College, Grahamstown (now Makhanda), SA, saw crystallography formally take off (Plug, 2020 ▸). Ogg then moved to the South African School of Mines and Technology (soon to become the University of the Witwatersrand) in Johannesburg from 1917 to 1919, and then to the University of Cape Town (UCT) from 1920 to 1936 (Plug, 2020 ▸; Schonland, 1948 ▸). He collaborated with W. H. Bragg (who often presented Ogg’s research at the Royal Society) and during this period published probably some of the earliest crystallography in South Africa (Ogg & Hopwood, 1916 ▸; Ogg, 1922 ▸, 1928 ▸). R. W. James was Ogg’s successor at UCT (James, 1948*a* ▸; James & Saunder, 1948 ▸) and collaborated with W. L. Bragg (Bragg *et al.*, 1922 ▸), and also produced a well-known textbook for the time (James, 1948*b* ▸). In the late 1940s, mainly under the influence of James, a school of X-ray analysis was started at UCT, which focused amongst others on the structure of aromatic molecules and molecular compounds using three-dimensional methods, extending the analyses to cover cases of molecular disorder (Bernal, 1962 ▸). James had a number of able assistants, among them Dr D. H. Saunder, Miss E. M. Archer (Mrs. D. H. Saunder), and Dr Aaron Klug (Nobel Prize for Chemistry, 1982), who did his first X-ray work in Cape Town (James, 1962 ▸). It was here at UCT that Klug began his studies on the use of Fourier transforms in crystal analysis, essential to his work on viruses.

During the following three decades, J. N. van Niekerk and a number of SA colleagues published early South African crystallographic work (van Niekerk, 1943 ▸; van Niekerk & Saunder, 1948 ▸; van Niekerk & Schoening, 1951*a* ▸,*b* ▸). The newly created National Physics Research Laboratory (NPRL; Kingwill, 1990 ▸), especially after F. H. Herbstein was additionally appointed, expanded the fundamental research (Harnik *et al.*, 1951 ▸, 1954 ▸; Herbstein & Schoening, 1955 ▸; Herbstein, 1951 ▸; Herbstein & Averbach, 1956 ▸; Gafner *et al.*, 1957 ▸). Shortly after that, J. C. A. Boeyens was also appointed at the NPRL and research accelerated (Boeyens & Herbstein, 1965 ▸, 1967 ▸; Roux & Boeyens, 1969 ▸; Verhoef & Boeyens, 1968 ▸; de Villiers & Boeyens, 1971 ▸), also when G. J. Kruger joined the laboratory (Kruger & Boeyens, 1968 ▸; Boeyens & Kruger, 1970 ▸). Three other sites in South Africa also developed after that, as manifested by significant inputs from L. R. Nassimbeni (UCT; Linck & Nassimbeni, 1973 ▸; Prout & Nassimbeni, 1966 ▸; Caira *et al.*, 1972 ▸, 1973 ▸), J. G. Leipoldt (University of the Orange Free State, Bloemfontein; Basson *et al.*, 1969 ▸; Bok *et al.*, 1970 ▸; Leipolt *et al.*, 1970 ▸; Leipoldt & Coppens, 1973 ▸; Wessels *et al.*, 1972 ▸) and J. S. Field (University of Natal; Einstein & Field, 1974 ▸; Field & Wheatley, 1972 ▸, 1974 ▸; Field *et al.*, 1974 ▸).

Mineralogy also expanded under J. N. van Niekerk (De Wet & van Niekerk, 1952–1953 ▸; Smuts *et al.*, 1966–1976 ▸) and J. P. R. de Villiers (de Villiers & Boeyens, 1971 ▸; de Villiers, 1971 ▸), with electron microscopy joining later (Auret *et al.*, 1978 ▸; Snyman & Neethling, 1983 ▸), as did protein work, paving the way for biological crystallographic studies (Greyling *et al.*, 1983 ▸; Retief *et al.*, 1984 ▸).

### 2.2. North/West Africa

Representative work in different fields of crystallography were produced in the latter decades of the 20th century by different researchers in Egypt, Tunisia, Algeria, and Morocco, following involvement of French universities at Bordeaux, Grenoble, Lille, Nancy, Paris, and Poitiers (Lecomte, 2021 ▸). Dorothy Hodgkin (Nobel Prize for Chemistry, 1964) was African born (Egypt) and contributed to the expansion of crystallography in North/West Africa. She was an honorary professor at the University of Ghana (1961–1962), and an honorary fellow of the Ghana Academy of Sciences (1965), and delivered a number of lectures during this period (Bodleian Libraries, 1993 ▸). Unfortunately, the coup in Ghana in 1966 severely delayed the further establishment of crystallography in Ghana.

Increased crystallograhic activities are illustrated by selected published work from the groups of K. El-Sayed (Egypt), H. Boughzala and M. Oumezzine (Tunisia), N. Benali-Cherif (Algeria), A. Thalal (Morocco) and co-workers (El Shazly & G. S. Saleeb, 1959 ▸; Lonsdale & El Sayed, 1965 ▸, 1966 ▸; Lonsdale *et al.*, 1966*a* ▸,*b* ▸; El-Sayed & Cosslett, 1977 ▸; El-Sayed *et al.*, 1986 ▸; Driss *et al.*, 1988 ▸; Bosio *et al.*, 1992 ▸; Duneau *et al.*, 1992 ▸; Boughzala *et al.*, 1993 ▸; Zeghdaoui *et al.*, 1994 ▸; Belam *et al.*, 1997 ▸; Bouhmaida *et al.*, 1997 ▸; Senis *et al.*, 1999 ▸). The ongoing struggle to access funding and instruments to expand crystallography in North Africa is reflected in a comment from Morocco (Thalal *et al.*, 2000 ▸).

### 2.3. Jan Boeyens and the IUCr in the 1990s

The IUCr Crystallography in Africa initiative was launched in 1999 following a proposal of former IUCr Executive Committee member Jan Boeyens from South Africa, with the aim of developing programmes and activities for the dissemination of crystallography on the African continent.

Part of this early initiative was the transfer of old (but still working) instruments into Africa, *e.g.*, to Nairobi, Kenya. The first was a single-crystal diffractometer donated by the CCDC while a second was a powder diffractometer from Boeyens’ lab (Levendis, 2007 ▸).

Prior to this, the establishment of the Indaba series in South Africa in 1995 by Boeyens created an additional portal for building out South African, and indeed African crystallography (Liles, 2015 ▸). These were supported by the IUCr Small Molecules Commission.

At the 17th IUCr Congress and General Assembly in Seattle in 1996, South Africa under the leadership of Jan Boeyens, Luigi Nassimbeni and Gert Kruger, launched an initiative to host the 19th IUCr Congress and General Assembly in 2002. South Africa lost the vote to Israel, and disappointingly this meeting was later moved to Geneva in Switzerland.

## 3. Period 2000 to 2013

**Figure 1:**
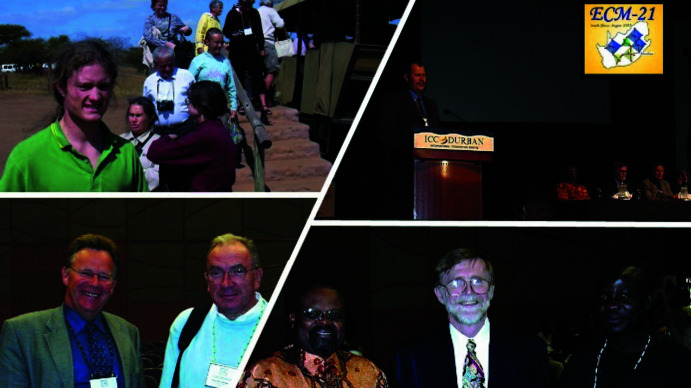
Clockwise from top left: ‘safari’ in a local game park; opening ceremony of ECM21 (Chair: A. Roodt); IUCr president W. Duax with the mayor of Durban (Ethekwini); ECA president C. Lecomte and delegates. (Photograph A. Roodt.)

Following the unsuccessful bid to host the IUCr Congress and General Assembly in 2002, upon the recommendation of P. Beurskens, Å. Oskarsson and C. Lecomte, South Africa (A. Roodt and J. Boeyens) prepared a bid to host the 21st European Crystallographic Meeting (ECM21) in Durban in 2003 (ECM, 2003) ▸; Roodt, 2003 ▸; Fig. 1 ▸), which turned out to be challenging but exiting.

The successful hosting of ECM21 paved the way for ECM24 to follow in Morocco; thus, two ECMs were hosted on the African continent is a short space of four years. Both these meetings ensured significant expanded interaction across Africa.

Following the success of ECM21, different crystallographic schools were initiated across Africa, including, to name a few, Morocco (L’EM3, 2006 ▸), South Africa (Nespolo *et al.*, 2010 ▸), Zimbabwe (Lecomte, 2011 ▸) and Ethiopia (Roodt, 2013 ▸).

Additional impetus for expanding North African crystallography is reflected in the first North African Conference in Morocco in November 2010, especially as it was attended by senior representatives of IUCr, ACA, AsCA and ECA (Kelly, 2011 ▸), as evidence of growing momentum towards developing crystallography in North Africa.

In addition, very importantly, during the period 2010–2013, a synchronized initiative to declare an International Year of Crystallography was launched. A. Thalal from Morocco, strongly supported by the IUCr (S. Larsen, President, IUCr, 2008–2011; and C. Lecomte, Vice President, IUCr, 2011–2014) and UNESCO (M. Nalecz and J. J.-P. Ngome Abiaga from the International Basic Sciences Programme), took the significant initiative to apply to the United Nations (IUCr, 2014*a* ▸ *b* ▸,*c* ▸). A concise timeframe for finally establishing an international year for crystallography was as follows (Thalal & Zema, personal communication):

**·**December 2010: start of the process for submitting the proposal for IYCr2013 through UNESCO.

**·**March 2011: after the Permanent Delegation of Morocco to UNESCO gave positive feedback, the process stops because time was too short.

**·**April 2011: start of the process again by submitting the proposal directly to the UN. Draft resolution is prepared and political action undertaken.

**·**November 2011: it is suggested to propose 2014 as IYCr (at this time, G. Desiraju is President and C. Lecomte Vice President of the IUCr).

**·**March 2012: draft resolution is submitted to the UN. The Moroccan Delegation succeeds in convincing six countries (Australia, Belgium, Luxembourg, Mexico, Poland and the Dominican Republic) to cosign the draft resolution and obtains unanimous support from the other delegations.

**·**July 2012: 2014 is proclaimed International Year of Crystallography, IYCr2014.

The successful declaration of IYCr2014 by the UN resulted in significant energy being injected into crystallography on a world-wide scale, and particularly in Africa. In 2013, M. Zema was appointed by the IUCr as Project Manager for the International Year of Crystallography (IYCr2014), becoming Outreach Officer in February 2015, and eventually Executive Outreach Officer in October 2015. Confirmation of the success of the International Year of Crystallography is manifested by much literature all around the world (IUCr, 2014*b* ▸).

As part of this initiative, A. Roodt was approached in 2013 by the then IUCr President, Gautam Desiraju, to host one of the three world summits in Africa, and proceeded with organizing IYCr2014 Africa under the title ‘Crystallography as a vehicle to promote science in Africa and beyond’. Significant initiatives in Africa flowed from this, as indicated in Section 4.

## 4. Period 2014–2019

**Figure 2:**
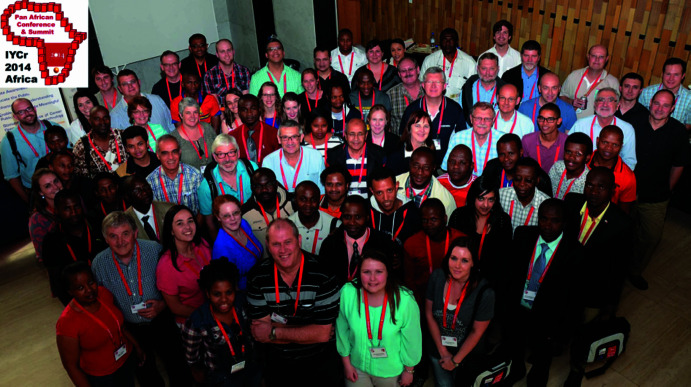
Delegates at IYCr2014 Africa in Bloemfontein, 2014. (Photograph A. Roodt.)

Supported by the South African Government, UNESCO and the IUCr, IYCr2014 Africa, the Pan-African Conference and Summit was hosted in Bloemfontein, South Africa, towards the end of 2014 (Roodt & Zema, 2014 ▸; IYCr2014 Africa ▸; Fig. 2 ▸). Delegates from more than twenty countries attended and discussions to expand initiatives on establishing AfCA commenced with more urgency.

Among the important initiatives flowing from IYCr2014 Africa was one to urge associated decision makers to (i) pro-actively continue programmes, ensuring the legacy of IYCr2014 and promotion of science was preserved and expanded, and (ii) over time provide basic diffraction equipment (but also other scientific infrastructure) for researchers in African countries to allow broader research in crystallography, see resolutions (Roodt & Zema, 2014 ▸).

**Figure 3:**
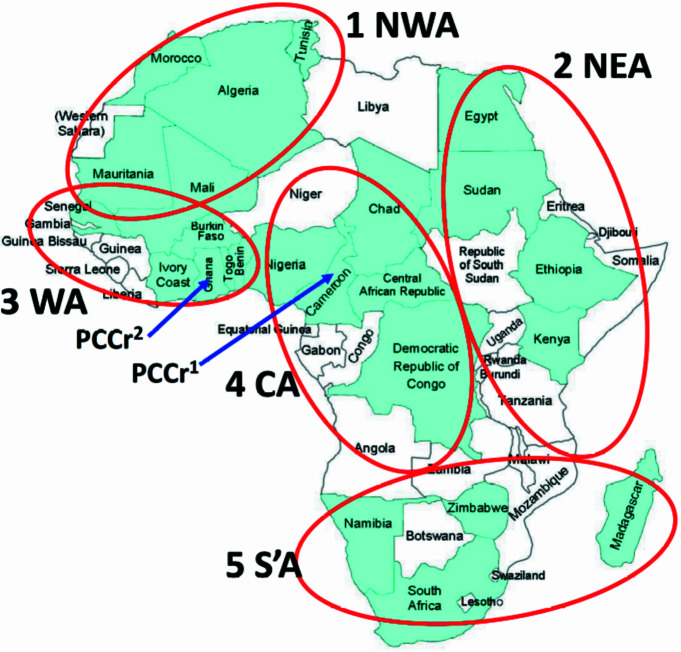
Division of Africa into different regions with champions in each region to coordinate and manage the appropriate division. NWA = North-West Africa, NEA = North-East Africa, WA = West Africa, CA = Central Africa, S’A = Southern Africa.

Moreover, the organisers committed to push forward with AfCA. Hence, IYCr2014 Africa enabled a focused action to establish an African Crystallographic Association, with many African research groups being represented. It was accordingly decided to divide Africa into different regions and identify regional champions to coordinate activities therein, see Fig. 3 ▸, and a very preliminary steering committee (A. Roodt, convenor) was assembled.

The preliminary steering committee of AfCA was unfortunately not very active for some months, and discussions were continued at the follow-up conference in Rabat, Morocco, to reflect on events of the International Year of Crystallography (IUCr, 2015 ▸), with the Executive Committee of the European Crystallographic Association (ECA) taking the lead (Bacchi *et al.*, 2017–2019 ▸; ECA/IUCr, 2019 ▸); A. Roodt (ECA President, 2012–2015) and A. Bacchi (ECA President (2015–2018).

Meanwhile, another thrust aimed at establishing an African Synchrotron emerged, which additionally provided impetus to an even broader community of African scientists, including crystallographers (AfLS, 2015–2023 ▸; Connell *et al.*, 2024 ▸).

During this period, the efforts of C. Lecomte and M. Zema continued with the IUCr–UNESCO OpenLab initiative and other actions across Africa (Zema & Lecomte, 2014–2023 ▸, 2014 ▸, 2015 ▸; Zema *et al.*, 2017 ▸), and should be recognized for significant contributions towards the expansion of crystallography and outreach in Africa. C. Lecomte as IUCr Executive Committee member and Vice President started with the first lectures in Dschang, Cameroon in 2013 (Bruker powder and single-crystal diffractometers also installed). This brought about the founding of the Cameroon Crystallographic Association, which was admitted as a member of the IUCr in 2014. Similarly, Morocco and Algeria were also admitted as members of the IUCr in 2014 (Haynes, 2023 ▸; IUCr, 2014*a* ▸). Tunisia was admitted as a member of the IUCr in 2017 (IUCr, 2017 ▸).

Following this, several IUCr–UNESCO OpenLabs were organized in *e.g*., Morocco, Ghana, Algeria, Tunisia, Kenya, Cameroon, Senegal, and Côte d’Ivoire (Zema & Lecomte, 2014–2023 ▸) with different partners, including Bruker, Malvern Panalytical, CCDC (Pradon, 2015 ▸), ICTP, ECA and others.

In 2018, the X-TechLab (2016 ▸, 2018 ▸), a crystallography hub for Western Africa, was established in Benin by the IUPAP–IUCr *LAAAMP* initiative founded by S. Mtingwa, M. Zema and S. Scandolo in 2016 (Zema *et al.*, 2016 ▸). Several training sessions, under the framework of the IUCr–UNESCO OpenLab initiative have since been organized there.

**Figure 4:**
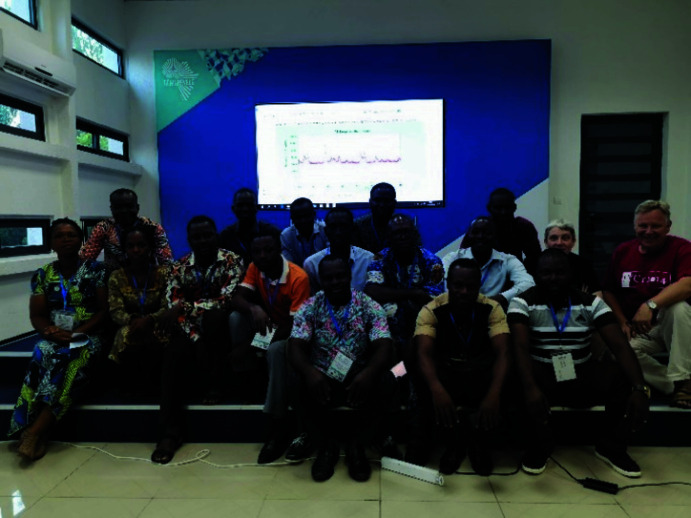
Researchers in Sèmè City, Benin, including F. Porcher (2nd row, 2nd from right) and C. Lecomte (2nd row, 1st from right). (Photograph courtesy C. Lecomte.)

Illustrated in the accompanying photo (Fig. 4 ▸) are researchers from X-TechLab, Sèmè City, Benin, celebrating a milestone, *i.e*., the first example of a molecule synthesized in Sub-Saharan/Central Africa for which the structure was also solved in Sub-Saharan/Central Africa.

**Figure 5:**
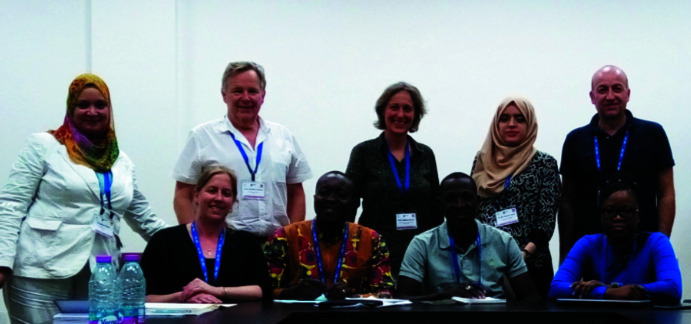
AfCA Steering Committee at the PCCr2 in 2018. Front row: Delia Haynes (South Africa), Chair; Patrice Kenfack Tsobnang (Cameroon), Secretary; Adam Bouraima (Gabon); Marielle Agbahoungbata (Benin). Back row: Seham Kamal (Egypt); Claude Lecomte (France), ex-officio member; Alessia Bacchi (Italy), ex-officio member; Rim Benali-Cherif (Algeria); Michele Zema (Italy), ex-officio member. (Photograph courtesy Rim Benali-Cherif.)

A more successful effort to formalize AfCA was then launched at the 1st Pan-African Conference on Crystallography in Dschang, Cameroon (IUCr, 2016 ▸), as well as at the IUCr–UNESCO Bruker OpenLab in Cote d’Ivoire (IUCr, 2018 ▸). Preliminary writing of the AfCA constitution and statutes commenced during this period (Bacchi *et al.*, 2017–2019 ▸), with the thrust gaining significant momentum and resulting in the AfCA Steering Committee being formally constituted at the 2nd Pan-African Conference on Crystallography in Accra, Ghana (PCCr2/AfLS2, 2019 ▸), see Fig. 5 ▸.

## 5. Period 2020–2023

During this next period, interaction within the African community to streamline and activate more colleagues continued. This was achieved despite Covid-19 by the online Pan-African Conference on Crystallography during November 2021 (ePCCR, 2021 ▸), which was well attended, and future activities regarding AfCA were further refined. AfCA was formally constituted at ePCCR. The 17 current AfCA members, which are also the founder members, are: Algeria, Benin, Cameroon, Congo, Côte d’Ivoire, Democratic Republic of Congo, Egypt, Gabon, Ghana, Kenya, Nigeria, Morocco, Rwanda, Senegal, South Africa, Tunisia and Zimbabwe (Haynes, 2023 ▸). The formation of AfCA was also ratified at the 33rd European Crystallographic Meeting in Versailles, France. (ECM33, 2022 ▸).

Final activities and all procedural matters were attended to at the second council meeting of AfCA at the 3rd Pan-African Conference on Crystallography in 2023 in Nairobi, Kenya (PCCr3, 2023 ▸). The formal constitution of AfCA, with D. Haynes as the first President, as the official fifth Regional Associate of the IUCr took place at the 26th Congress and General Assembly of the International Union of Crystallography in Melbourne, Australia, 22–29 August 2023 (IUCr, 2023 ▸).

## 6. Future prospects

Challenges for AfCA once formally inaugurated certainly lie ahead, amongst others to expand African science significantly. Using crystallography as a multi-disciplinary vehicle, decisive and targeted actions are required, taking into account the ever-growing population of the continent (more than 50 countries, population over 1.3 billion), compared to Europe (44 countries, over 740 million inhabitants). Moreover, a concerted effort is necessary to pro-actively involve even more work from the material science, geology, mineralogy, physics, electron microscopy, African Lightsource and biology/protein communities.

It is, however, also incredibly important that the IUCr as custodian and the ECA continue to pro-actively interact with AfCA, and that the science ministries in all African countries become significantly more involved to enable AfCA to contribute not only to challenges directly associated with Africa, but also to the world, on issues such as pandemics and global warming.

It is impossible to acknowledge everyone who has contributed to an effort such as this, and to finally observe the birth of AfCA. Nevertheless, the tireless efforts and support from the IUCr, ECA and UNESCO, funding agencies across the globe, the private sector and government funding are gratefully acknowledged. Positive inputs from the reviewers of the original manuscript and the editor to the final paper are also gratefully acknowledged. However, in particular, all scientists involved for more than a century, and specifically all students appreciating the wonder of crystallography, are thanked and encouraged to do even better science and become even more involved. Crystallography is an ideal vehicle for this.

## Figures and Tables

**Table 1:** Selected important time periods and events *en route* to the formation of AfCA; references are given in the main text

Period/Year	Activities/outcomes	Some principal driver(s)/role players
pre-1999	Activate Research in Crystallography	IUCr/Académie across Africa
1996	Attempt to host IUCr General Assembly in SA	Boeyens (IUCr)
1999	*Launch of the IUCr ‘Crystallography in Africa’ initiative.*	Boeyens (IUCr), IUCr EC.
2002-	Moroccan & Algerian Crystallographic Associations founded.	Lecomte, Fuess (ECA).
2003	ECM21, Durban, South Africa.	Lecomte, Roodt (ECA).
2007	ECM24, Marrakech, Morocco.	Lecomte, Fuess, Thalal (ECA).
2010	1st North African Crystallographic Conference, Casablanca, Morocco.	El Jazouli, Thalal, others (AMC); Larsen, Lecomte (IUCr); Garcia Granda (ECA).
2010-13	Preparatory work and launch of the International Year of Crystallography	Thalal, Lecomte, Larsen, Desiraju, Zema (IUCr); Nalecz, Ngome-Abiaga (UNESCO IBSP)
2014	IYCr2014 Pan African Summit Meeting, Bloemfontein, South Africa. *Formulate idea of an AfCA; Steering Committee nominated*	Roodt (ECA; nominated chair of AfCA SC); Zema, Thalal, Desiraju (IUCr); Nalecz, Ngome Abiaga (UNESCO); Nyanganyura (ICSU-ROA)
2014–18	10+ IUCr-UNESCO OpenLabs (Algeria, Benin, Cameroon, Côte d’Ivoire, Ghana ×2, Kenya, Morocco, Senegal ×2, Tunisia)	Lecomte, Zema, Desiraju (IUCr); Nalecz, Ngome Abiaga (UNESCO); industrial partners (Bruker, CCDC, Malvern Panalytical, others), institutional partners (ECA, ICTP, others)
2015	‘Crystallography Matters More’ conference, Rabat, Morocco	Thalal, Zema, Lecomte (IUCr); Roodt (ECA, AfCA)
2015	ICSU grant ‘Building science capacity in Africa *via* crystallography’ awarded	Roodt (ECA); Zema (IUCr)
2016	PCCr1, Dschang, Cameroon	Lecomte, Kenfack, Tonle, Ngoune, Bacchi, others; IUCr, ECA, AfCA
2016	ICSU grant ‘Utilization of Light Source and Crystallographic Sciences to Facilitate the Enhancement of Knowledge and Improve the Economic and Social Conditions in Targeted Regions of the World’ awarded; *LAAAMP* is founded	Zema (IUCr); Mtingwa, Scandolo (IUPAP); *LAAAMP*
2017	Workshop ‘Crystallography in emerging nations: projects for a sustainable development of education and research infrastructure in Africa’, 24th IUCr Congress, Hyderabad, India; *AfCA strategic regions identified and representatives nominated*	Zema, Lecomte (IUCr). Roodt, Haynes, all members of the AfCA SC; Bacchi, Lecomte, Zema
2018	Foundation of X-TechLab, Sèmè City, Benin	Zema (IUCr, *LAAAMP*); Mtingwa, Scandolo (IUPAP, *LAAAMP*); D’Almeida (X-TechLab)
2019	PCCr2, joint with AfLS2, Accra, Ghana; *new chair and members of AfCA Steering Committee nominated.*	Lecomte (IUCr); Haynes (ECA, AfCA); Artioli, Haynes (nominated chair of AfCA SC), all members of the AfCA SC, Bacchi, Lecomte, Zema
2021	ePCCr, joint with AfLS and AfPS, hosted by X-TechLab, Benin; *17 Nov 2021: formal constitution of AfCA. 1st AfCA Executive Committee nominated*	Agbahoungbata, D’Almeida (X-TechLab, *LAAAMP*); Mehlana (AfCA); Connell, Norris (AfLS); Wague (AfPS). Haynes, Kenfack, all members of the AfCA EC, Bacchi, Lecomte, Zema; IUCr, ECA
2022	ECM33, Versailles, France. AfCA ExComm; *Formation of AfCA is ratified by ECA*	Haynes, Kenfack, Lecomte, AfCA, ECA, IUCr
2023	PCCr3, Nairobi, Kenya	Amolo *et al.*; AfCA, IUCr, ECA
2023	IUCr2023, Melbourne, Australia, Aug 2023; *AfCA EC present FINAL PROPOSAL to IUCr GA; AfCA is admitted as a Regional Associate of the IUCr*	Haynes, all members of AfCA EC
